# Development of Reverse Transcription Loop-Mediated Isothermal Amplification Assay for Rapid and On-Site Detection of Avian Influenza Virus

**DOI:** 10.3389/fcimb.2021.652048

**Published:** 2021-04-19

**Authors:** Mohsen Golabi, Marion Flodrops, Beatrice Grasland, Aaydha C. Vinayaka, Than Linh Quyen, Trieu Nguyen, Dang Duong Bang, Anders Wolff

**Affiliations:** ^1^ Laboratory of Applied Micro and Nanotechnology (LAMINATE), Department of Biotechnology and Biomedicine, Technical University of Denmark, Lyngby, Denmark; ^2^ Laboratory of Ploufragan-Plouzané-Niort, Unit of Avian and Rabbit Virology, Immunology and Parasitology, French Agency for Food, Environmental and Occupational Health & Safety (ANSES), Ploufragan, France; ^3^ BioLabChip Group, Department of Biotechnology and Biomedicine, Technical University of Denmark, Kongens Lyngby, Denmark

**Keywords:** Avian influenza virus, on-site detection, RT-LAMP, RT-PCR, colorimetric visualization, rapid detection

## Abstract

Avian influenza virus (AIV) outbreaks occur frequently worldwide, causing a potential public health risk and great economic losses to poultry industries. Considering the high mutation rate and frequent genetic reassortment between segments in the genome of AIVs, emerging new strains are a real threat that may infect and spread through the human population, causing a pandemic. Therefore, rapid AIV diagnostic tests are essential tools for surveillance and assessing virus spreading. Real-time reverse transcription PCR (rRT-PCR), targeting the matrix gene, is the main official standard test for AIV detection, but the method requires well-equipped laboratories. Reverse transcription Loop-Mediated Isothermal Amplification (RT-LAMP) has been reported as a rapid method and an alternative to PCR in pathogen detection. The high mutation rate in the AIV genome increases the risk of false negative in nucleic acid amplification methods for detection, such as PCR and LAMP, due to possible mismatched priming. In this study, we analyzed 800 matrix gene sequences of newly isolated AIV in the EU and designed a highly efficient LAMP primer set that covers all AIV subtypes. The designed LAMP primer set was optimized in real-time RT-LAMP (rRT-LAMP) assay. The rRT-LAMP assay detected AIV samples belonging to nine various subtypes with the specificity and sensitivity comparable to the official standard rRT-PCR assay. Further, a two-color visual detection RT-LAMP assay protocol was adapted with the aim to develop on-site diagnostic tests. The on-site testing successfully detected spiked AIV in birds oropharyngeal and cloacal swabs samples at a concentration as low as 10^0.8^ EID_50_ per reaction within 30 minutes including sample preparation. The results revealed a potential of this newly developed rRT-LAMP assay to detect AIV in complex samples using a simple heat treatment step without the need for RNA extraction.

## Introduction

Influenza viruses have a negative-sense single-stranded RNA genome, and are classified in four genera, *Alphainfluenzavirus*, *Betainfluenzavirus*, *Deltainfluenzavirus*, and *Gammainfluenzavirus* under the Orthomyxoviridae family. Each Genus only contains one species or type named Influenza A, B, C, and D respectively. Type A influenza virus infects birds and some mammalian species including humans and it is responsible for all flu pandemics (Dhumpa et al., 2010). Influenza A viruses are host-adapted and the subtypes that infect avian species are distinct from the subtypes that infect humans. However, on rare occasions, a virus may change enough to infect other species and circulate in a new host and infect other species. Influenza A viruses are classified according to their surface glycoproteins (hemagglutinin, HA, and neuraminidase, NA). Sixteen hemagglutinin (H1–H16) and nine neuraminidase (N1–N9) subtypes have been reported in birds and may occur in any combinations. The segmented nature of the AIV RNA genome involved genetic evolution derived by mutations or re-assortments (Webster et al., 1992). As a rare event, H5 or H7 subtypes are able to evolve from Low Pathogenic AIV (LPAIV) to high pathogenic AIV (HPAIV) by mutation (nucleotide substitutions or insertions) in their HA sequence. LPAIV can mutate and change to an HPAIV strain while circulating among poultry (Bodewes and Kuiken, 2018). Some AIV strains can transmit from bird to human through direct contact with infected birds or contaminated environments but direct transmission of AIV from human to human is a rare event (Abdel-Ghafar et al., 2008). As some genes of AIVs appeared in the genomes of three human influenza strains that caused pandemics in the 20^th^ century, it is thought that the pandemic influenza strains had a link with AIV (Euro Surveill, 2007). Serological evidence shows that many LPAIV outbreaks have not been recognized in domestic poultry as it has a mild effect on infected birds. This infers that the rate of LPAIV outbreaks are more common in birds than the numbers reported by the authorities (Capua and Alexander, 2004; Tweed et al., 2004; Puzelli et al., 2005). HPAIV/LPAIV re-assortments are able to generate new viruses and trigger a new outbreak (Olsen et al., 2006). Wild birds are a natural reservoir of AIVs and responsible for transmission to poultry farms (Clark and Hall, 2006). Industrial poultry around the world frequently faces HPAIV outbreaks and suffers economic losses. Eradication of AIV in the natural reservoir is impossible. Therefore, AIV surveillance including early and accurate detection in wild birds and poultry farms is a critical measure to control the spreading of the virus and to reduce the risk of emerging new strains. Different methods such as cultivating in embryonated chicken eggs, serological based assays, as well as nucleic acid amplification techniques have been developed for AIV detection. Real-time reverse transcription polymerase chain reaction (rRT-PCR) assay targeting Matrix gene (M-gene) of AIV has been used as the official standard test by reference laboratories to detect the AIVs. The rRT-PCR assay can detect AIV with excellent specificity and sensitivity even better than the culture test, with an improved detection rate of 2-13% (WHO, 2005). However, this expensive assay is only suitable for well-equipped laboratories and requires trained staff. Rapid detection is a crucial step to control AIV outbreaks. On-site detection tests will provide faster results by omitting the sample-to-lab transporting time and are easy to perform by non-trained staff. Commercially available rapid human influenza diagnostic tests, mostly based on antigen/antibody detection tests in a paper-based platform, provide results within 30 minutes and are easily adaptable for rapid AIV detection on-site. However, these tests have low sensitivity (70-75%) and moderate specificity (90-95%) that are not satisfactory. The Positive Predictive Values of 79-87% and the Negative Predictive Values of 39-75% of these rapid tests are significantly lower than that of the gold standard tests (WHO, 2005). Loop-Mediated Isothermal Amplification (LAMP) is an isothermal nucleic acid amplification technique carried out at 60-65°C. The test uses a polymerase with high strand displacement activity and three or four pairs of primers, which identify six or eight distinct regions on the target gene. The primer set consists of two outer primers and two inner primers; a forward inner primer (FIP) and a backward inner primer (BIP). As a result of employing four primers that must hybridize to the six distinct regions on the target sequence to initiate the amplification, LAMP has been reported as an equal or more sensitive and specific amplification technique than regular PCR (Bruce et al., 2015). In contrast to PCR, which generates a single size DNA product, positive LAMP reaction results in a massive production of DNA of varying sizes. This high DNA production feature of LAMP facilitates the ability to distinguish positive and negative results, even by the naked eye (Mori et al., 2001; Arunrut et al., 2016). LAMP has been reported to be more resistant to inhibitors that ease the sample preparation steps (Francois et al., 2011). Having several advantages such as being simple to operate, rapid, resistant to inhibitors, cost efficient, easily detectable results, and high sensitivity and specificity, LAMP is an ideal nucleic acid amplification technique to be adapted for on-site, rapid, diagnostic assays. Although employing four/six primers provides very high specificity features to LAMP, it also makes it challenging to design a set of LAMP primers for the target genes containing high mutation rates, such as the AIV genome. On the other hand, the specificity of a designed primer set might decrease over time due to emerging and accumulation of point mutations in a dynamic target like AIV genome. As an example, the EU AI Diagnostic Manual published in 2006, recommended an rRT-PCR assay targeting M gene for AIV detection. The assay had already been validated by a blind ring trial assessment in 2004. But after only five years it was reported to have relatively low sensitivity for detecting two new AIV clades emerged in 2009 (Slomka et al., 2012). Designing a primer set based on the latest isolated AIVs that have a highly mutable genome is therefore essential. The influenza virus A genome consists of eight single-stranded RNA segments including matrix gene segments with approximately 1 k bases. In this study, 800 M-gene sequences of newly isolated AIVs were collected from AIV Eurasian lineages for designing a highly sensitive and specific LAMP primer set. We tested the specificity, sensitivity, and simplicity to operate on this newly developed rRT-LAMP and compared against reference rRT-PCR stated in the EU AI Diagnostic Manual. Then the newly designed primer set was adapted into a commercial LAMP test for on-site testing applicable to perform a rapid and accurate AIV detection.

## Materials and Methods

### Virus Samples

Ten reference inactivated AIV strains and three non-Influenza avian virus strains (**Table 1**) as well as 14 AIV samples isolated from wild birds in Denmark in 2018 were used to evaluate the newly developed RT-LAMP method.

**Table 1:** The list of Virus strains used in this study.

Virus sample	Subtype	Location of isolation	Collection time	Host	Virus reference
Avian influenza	H5 N2	France	2015	Duck	150233b/3.1ib
Avian influenza	H5 N2	France	2005	Duck	05057b3.4i
Avian influenza	H5 N3	France	2002	Duck	02166/4.4ia
Avian influenza	H7 N7	France	2010	Mallard	100365e/3.1i
Avian influenza	H7 N1	England	1979	Starling	983/1979/5.10i
Avian influenza	H7 N1	France	2010	Mallard	06159/4.2i
Avian influenza	H9 N2	USA	1966	Turkey	Wisconsin/c/3.2i a
Avian influenza	H6 N8	France	2009	Pekinduck	090173/4.1i
Avian influenza	H1 N1	Canada	1976	Duck	Alberta/35/76/3.3ia
Avian influenza	H3N1	France	2019	Chicken	D1902689/lot 2.1i
Avian Paramyxovirus	PMV1	USA	1956	Chicken	LaSota/4.10i
Avian Paramyxovirus	PMV2	USA	1946	Chicken	Yucaipa/1956_6.1i
Avian Paramyxovirus	PMV7	USA	1975	Dove	Tennessee/4/1975_4.1i

### AIV RNA Extraction

Total RNA was extracted using a purification Kit (Norgen Biotek, Canada) according to the manufacturer’s instructions. Briefly, 350 µl of RL buffer was added into 100 µl of sample. The sample was mixed by vortex for 15 sec at room temperature. Then 200 µl of ethanol (96-100%) was added and mixed. The mixture was pipetted into a spin column and centrifuged at 3500 g for 1 min. Four hundred µl of a wash solution were added to the spin column and centrifuged at 14.000 g for 1 min. The washing step was repeated three times. Thereafter, 50 µl of the elution buffer was added to the spin column in an eluent collection tube and centrifuged at 200 g for 2 min. The eluent of the extracted RNA was collected and used in the downstream tests or stored at -80°C until use.

### Real-Time RT-PCR Reference Method for AIV Detection

The Spackman rRT-PCR assay to detect the M-gene of AIV was performed according to the reference protocol provided by Avian Influenza Community Reference Laboratory https://science.vla.gov.uk/flu-lab-net/docs/pub-protocol-ai-vi493.pdf. The rRT-PCR was performed using Qiagen (QIAGEN, Hilden, Germany) one-step RT-PCR kit according to the manufacturer’s instructions. The final 25 µl volume of the rRT-PCR reaction mixture contained 13.75 µl of DEPC treated water, 5 µl of 5x Qiagen 1-step RT PCR buffer, 0.375 µl of Rox ref dye (pre diluted 1:500 in DEPC water), 1 µl of dNTPs mix, 1.25µl of Magnesium chloride 25mM, 0.1µl of RNAsin (40U/µl), 1µl of Qiagen 1 step RT PCR enzyme mix, 2 µl of each 50µM Forward primer, Reverse primer and 2.5 µl of 30µM hydrolysis probe labeled with FAM and TAMRA and 2 µl of sample RNA. The DEPC water was used as a no template control. The rRT-PCR conditions were 50°C for 30 min for cDNA synthesis followed by 15 min at 95°C to activate the hot start polymerase enzyme. The PCR conditions include 40 cycles of 95°C for 10s and 60°C for 20s. Fluorescence data were acquired at the end of each annealing step.

### Design LAMP Primers for AIV Detection

To design an efficient LAMP primer for AIV detection, sequences of whole matrix gene of 800 AIV strains, that were isolated and collected in Europe between 2010 and 2018 and obtained from Global Initiative on Sharing All Influenza Data (GISAID), were aligned using UGENE, the free open-source, cross-platform bioinformatics software. The consensus sequence was used to design the LAMP primer using online software Primer Explorer V5 (http://primerexplorer.jp/lampv5e/index.html). Highly conserved regions on the consensus sequences of the M gene were manually specified for designing primers using PrimerExplorer V5 software.

### Sample Preparation for AIV RT-LAMP Assay

Heat lysis method was used in the sample preparation step for rRT-LAMP and on-site testing assays. Inactivated AIV samples were diluted in RNAse free water or spiked in the real matrix and incubated at 95°C for 5 min to lyse the host cells and the virus particles to release the genomic RNA content from the virus capsid protein. This heat-treated sample was added directly to the rRT-LAMP and on-site testing tube.

### Real-Time RT-LAMP Assay

The rRT-LAMP reaction mixture includes LAMP primers (**Table 2**) with the final concentration of 0.2 µM of each F3/B3; 1.6 µM of each FIP/BIP; 0.8 µM of each LF/LB; 0.5 M Betaine; 1X isothermal amplification buffer (BioLabs Inc. New England); 1.4 mM of mix dNTPs; 5 µM of SYTO 9; 8U of WarmStart *Bst*2 DNA polymerase and 6U of WarmStart RTx reverse transcriptase (BioLabs Inc. New England). 2 µl of heat-lysed sample was added to 18 µL of the rRT-LAMP reaction mixture and incubated in a thermocycler at 65° C for 60 minutes followed by 1 minute at 95°C for heat inactivation of both enzymes. Fluorescence data were acquired every minute during the isothermal amplification step. After LAMP amplification, 2 µL of the amplified product was loaded on 2% agarose gel and electrophoresis for 60 minutes at 100 Volts in the Tris-acetate EDTA buffer. The gel was stained with SYBR safe, and gel images were acquired using a gel-documentation system (Gel Doc 2000, Bio-Rad) visualized under UV light.

**Table 2:** List of RT-LAMP primers and sequences (F3, B3, FIP, BIP, LF, and LB) for AIV detection and the primers (Forward and Reverse) and probe oligonucleotides recommended by the reference protocol for AIV detection by rRT-PCR.

Primer or probe	Sequence (5’ to 3’)	Location
F3	GCAGGTAGATATTGAAAGATGAGTC	8-33
B3	CTCACTGGGCACGGTGA	223-239
FIP	GGCTTTGAGGGGGCCTGA-TTCTAACCGAGGTCGAAACG	F1 (75-92), F2 (34-53)
BIP	CTTGAAGATGTCTTTGCAGGGAAGAACA-TAGTCAGAGGTGACAGGATTGG	B1 (107-135), B2 (174-195)
LF	CGGGACGATAGAGAGAACGTA	54-74
LB	CGAGGCTCTCATGGAATG GCTAAAG	142-167
Forward	AGATGAGTCTTCTAACCGAGGTCG	19-47
Reverse	TGCAAAAACATCTTCAAGTCTCTG	101-125
Probe	TCAGGCCCCCTCAAAGCCGA	75-89

The nucleotide position is corresponding to the Influenza A virus (A/African starling/England-Q/983/1979(H7N1)) matrix protein genes sequence of the 5’ end.

### AIV On-Site Testing

For on-site testing, a commercial universal LAMP kit Warm-Start Colorimetric LAMP 2X Master Mix (BioLabs Inc. New England) was used. The newly designed AIV LAMP primer set was added to the 2X Warm-Start Colorimetric LAMP Master Mix followed by RNAse free water to reach the final 1X concentration of the reaction mixture. For the on-site test, 2µL of heat-lysed sample was added to the test tube including 18 µL of reaction mix and incubated in a heat block for 25 minutes in 65°C. The test is considered positive if the original pink color of the testing tube shifts to yellow.

### Specificity of AIV rRT-LAMP Assay

To evaluate the specificity of the designed LAMP primers and rRT-LAMP assay, 23 AIV samples and three non-influenza avian viruses belonging to *Paramyxoviridae* family were used in parallel with rRT-PCR Assay.

### Sensitivity of AIV-RT LAMP Assay

The analytical sensitivity of the rRT-LAMP methods was determined and compared with the On-site test and rRT-PCR using inactivated influenza A virus H3N1_A/chicken/France/D1902689/2019 titrated at 10^8.5^ EID_50_/mL sample. A serial 10-fold dilution (10^8.5^ to 10^0.5^ EID_50_/mL) of the sample was prepared in RNAse free water and used in these parallel assays. For the rRT-PCR, total RNA is prepared as described above while for rRT-LAMP and on-site LAMP test, the sample prepared by heat lysis method as described before. The sensitivity experiments are repeated in at least three replicates.

### Virus Spiked Real Matrix Sample

The inhibition effects of real matrix samples on the rRT-LAMP and the on-site LAMP tests were examined. Oropharyngeal and cloacal swabs taken from chickens were resuspended in PBS. Chicken organs including, Larynx, Liver, Pancreas, Heart, Spleen, Brain and Lung were obtained from the fresh chicken carcass (acquired from a local market in Copenhagen). The samples (100 mg/mL) in PBS were homogenized, and used as real organ matrix samples. The matrix was used as a negative control in rRT-LAMP and on-site LAMP test and then 90µL of the samples were spiked with 10 µL of high (10 ^8.5^ EID_50_/mL) or low (10 ^4.5^ EID_50_/mL) concentration inactivated AIV reference sample.

## Results

### AIV Matrix-Gene Alignment and LAMP Primer Designing

**Figure 1:**
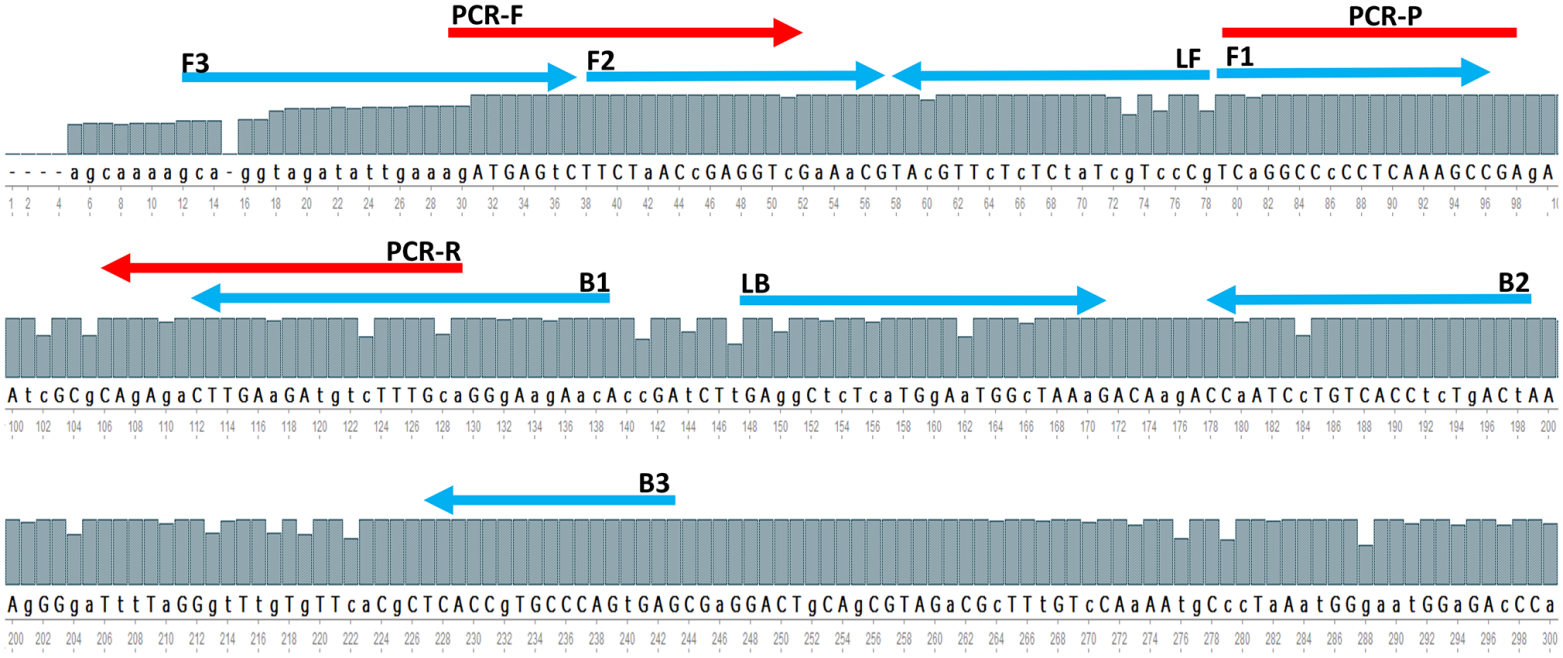
A The partial consensus sequence of AIV M-gene and the location of designed RT-LAMP primers (blue arrow) and referenced method rRT-PCR primers and probe (red arrow).

The whole Matrix gene sequences of 800 AIV strains acquired from GISAID were aligned with the UGENE program (http://ugene.net/) to find the sequence of relatively conserved regions on recently circulating AIVs. The alignment results revealed many point mutations in the entire M-gene segment, although the M-gene is the best conserved gen in AIV. Therefore, the most relative conserved location is manually defined using the online software PrimerExplorer V5 (http://primerexplorer.jp/lampv5e/index.html), for primer designing. **Figure 1** showed the locations of the designed LAMP primers and the primers and probes for rRT-PCR as suggested by the reference protocol for AIV M-gene detection. The oligonucleotide sequences of primers and the probes are shown in **Table 2**. Preliminary experiments are performed only with primers in the absence of any templates in order to evaluate the negative control precision revealed no significant primer dimer or self-amplifying reaction.

### Specificity Test

**Figure 2:**
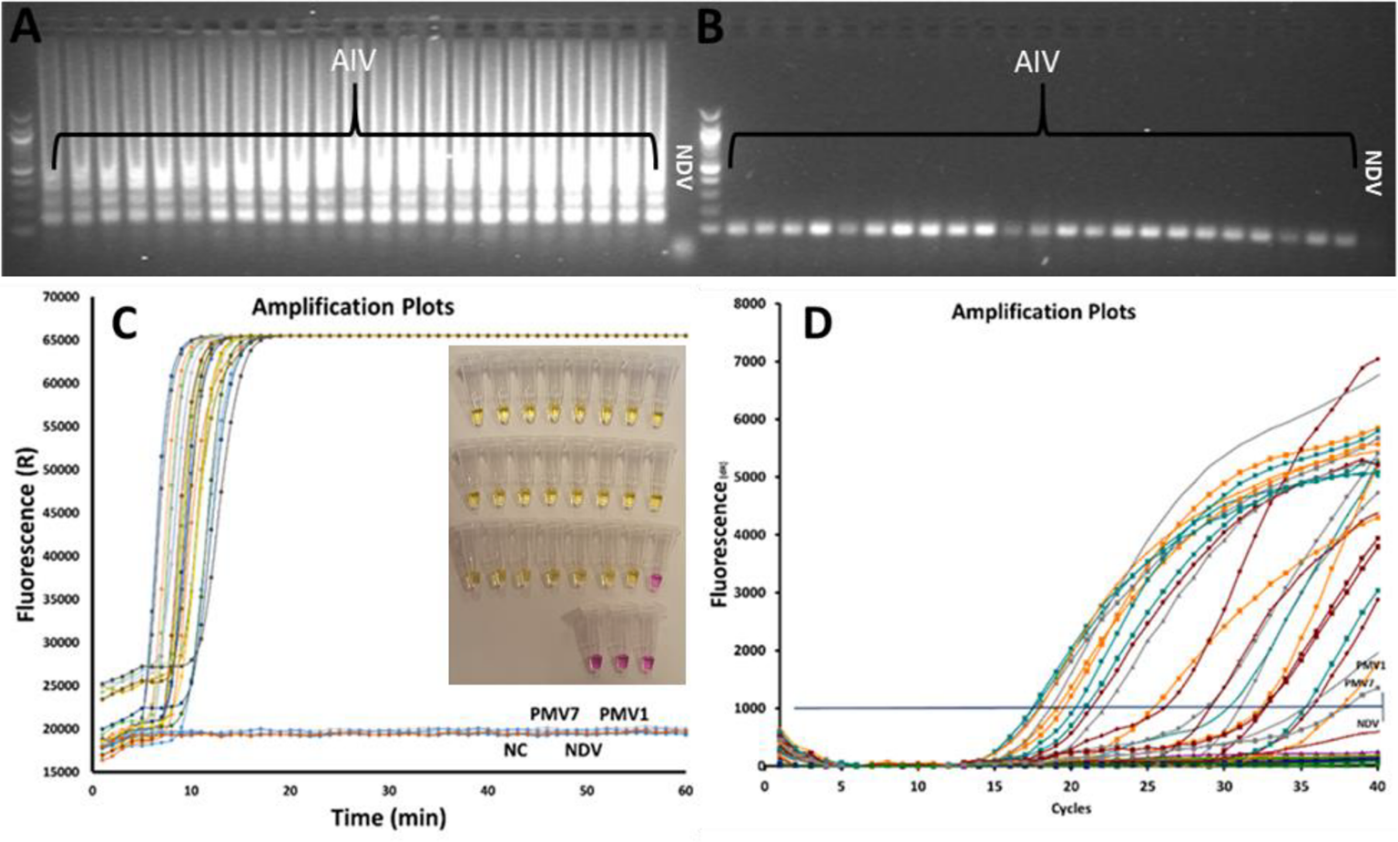
Comparing rRT-LAMP, on-site test and rRT-PCR methods for AIV detection, 23 AIV strains and 3 non-AIV avian strains were tested. The gel electrophoresis image of rRT-LAMP **(A)** and RT-PCR products **(B)**. The amplified curves in rRT-LAMP **(C)**. The results of the on-site test (insert **Figure** in **C**) and the rRT-PCR amplification curve **(D)**.

A total of 23 AIV subtypes are used to test the specificity of the AIV RT-LAMP. The results of the experiments are shown in **Figure 2**. The tests were performed in three parallel experiments using rRT-LAMP (**Figure 2C**), on-site testing [**Figure.2C** insert image (color change)] and rRT-PCR (**Figure 2D**). The results of the experiments are shown in **Figure 2**. Both rRT-LAMP and on-site test methods could successfully detect the virus in all the AIV samples (**Figure 2C**) while no LAMP amplified product was observed in the samples with non-AIV and negative control samples. The gel electrophoresis analysis (**Figures 2A, B**) of reaction mixture confirmed the results.

### Sensitivity of rRT-PCR, rRT-LAMP and On-Site LAMP Testing

**Figure 3:**
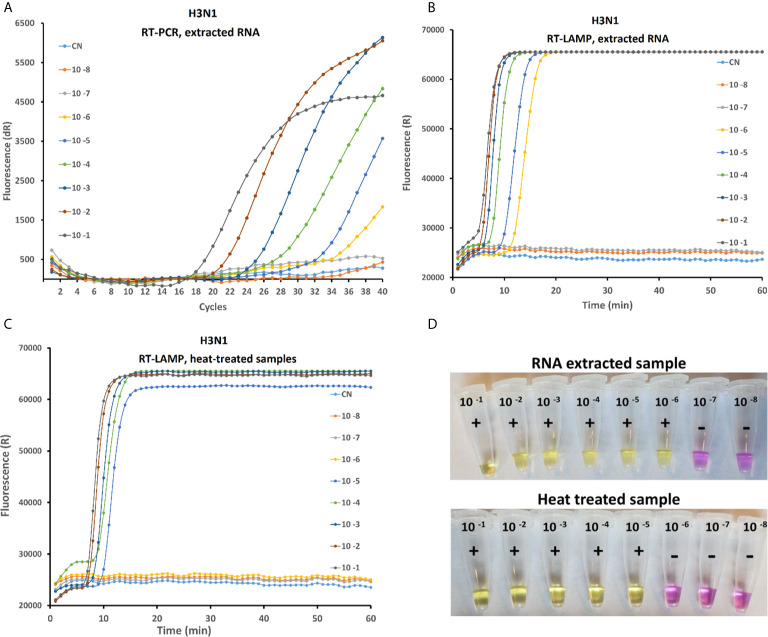
Comparative study of the sensitivity of rRT-PCR, rRT-LAMP and on-site testing methods on serial 10-fold dilution of the H3N1 AIV sample with an initial viral titer of 10 ^8.5^ EID_50_/mL. The RT-PCR amplification curve with purified RNA **(A)** RT-LAMP amplification curved with purified RNA **(B)** and with heat treated sample **(C)** and Onsite LAMP test using RNA extracted (**D** up) and heat lysed samples (**D** down).

Sensitivity of the rRT-LAMP was compared with RT-PCR and on-site test assay (**Figure 3**) in order to evaluate the performances of the three methods. For the rRT-PCR, the total RNA is extracted from serial 10-fold diluted AIV samples as explained above. In rRT-PCR, the reaction was positive up to 10^-6^ dilution of AIV sample (10^-0.2^ EID_50_ per reaction). Negative results were obtained when testing the 10^-7^ dilution sample or a C_t_ value above 36 was observed in some repeats, which is not considered as positive according to the standard protocol. Analytical sensitivity of rRT-LAMP and on-site test method similar to rRT-PCT (**Figure 3B**) when purified RNA was used as a template. However, the sensitivity of rRT-LAMP and on-site test was 10 fold lower with test samples prepared by heat-treated method (no RNA purification) (**Figures 3C, D**).

### Detection of AIV in Virus Spiked Real Matrix Samples

**Figure 4:**
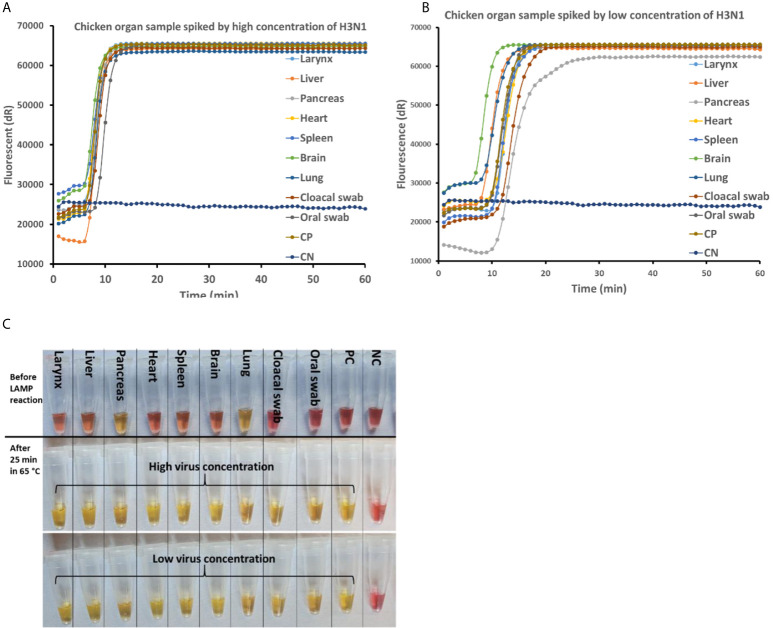
AIV detection in real matrix samples with rRT-LAMP assay and on-site testing method. The real matrix samples (90µL) are spiked with 10 µL of high (10 ^8.5^ EID_50_/mL) and low (10 ^4.5^ EID_50_/mL) concentrations of AIV H3N1. The samples are heat treated at 95°C for 5 min and subjected to rRT-LAMP and on-site testing. rRT-LAMP amplification curve with high virus concentration **(A)** and low virus concentration **(B)**. **(C)** Before (top) and after LAMP amplification with high (middle) and low (bottom) concentration of AIV spiked in real matrix samples tested using on-site method.

The newly developed rRT-LAMP and on-site test methods were tested with simulated real samples spiked with AIV samples. As shown in **Figure 4A**, no effect of real sample matrix on the rRT-LAMP reaction was observed at high concentration of the target templates (10 ^7.5^ EID_50_/mL). While at lower concentration (10 ^3.5^ EID_50_/mL) of the target template, (**Figure 4B**), there were noticeable variations in the Tt (Threshold time) among different matrix types, however, the difference was not significant enough to affect the assay performances.

## Discussion

Increase in the human populations in recent decades directed the agriculture system to increase food production, such as the growing of industrial poultry production in many countries. Birds are breeding in elevated densities in industrial farms, facilitating the transmission of infectious pathogens such as AIV. An easy to perform on-site test with similar sensitivity to rRT-PCR is an urgent need for rapid detection of AIV (Nguyen et al., 2020). LAMP has been reported as an accurate pathogen detection method suitable for on-site testing (Chow et al., 2020; Wu et al., 2020; Yin et al., 2020). **Scheme 1** shows the process and the performing time of the current rRT-PCR method compared with the on-site testing method developed in this study based on the LAMP principle.

**Schematic 1:**
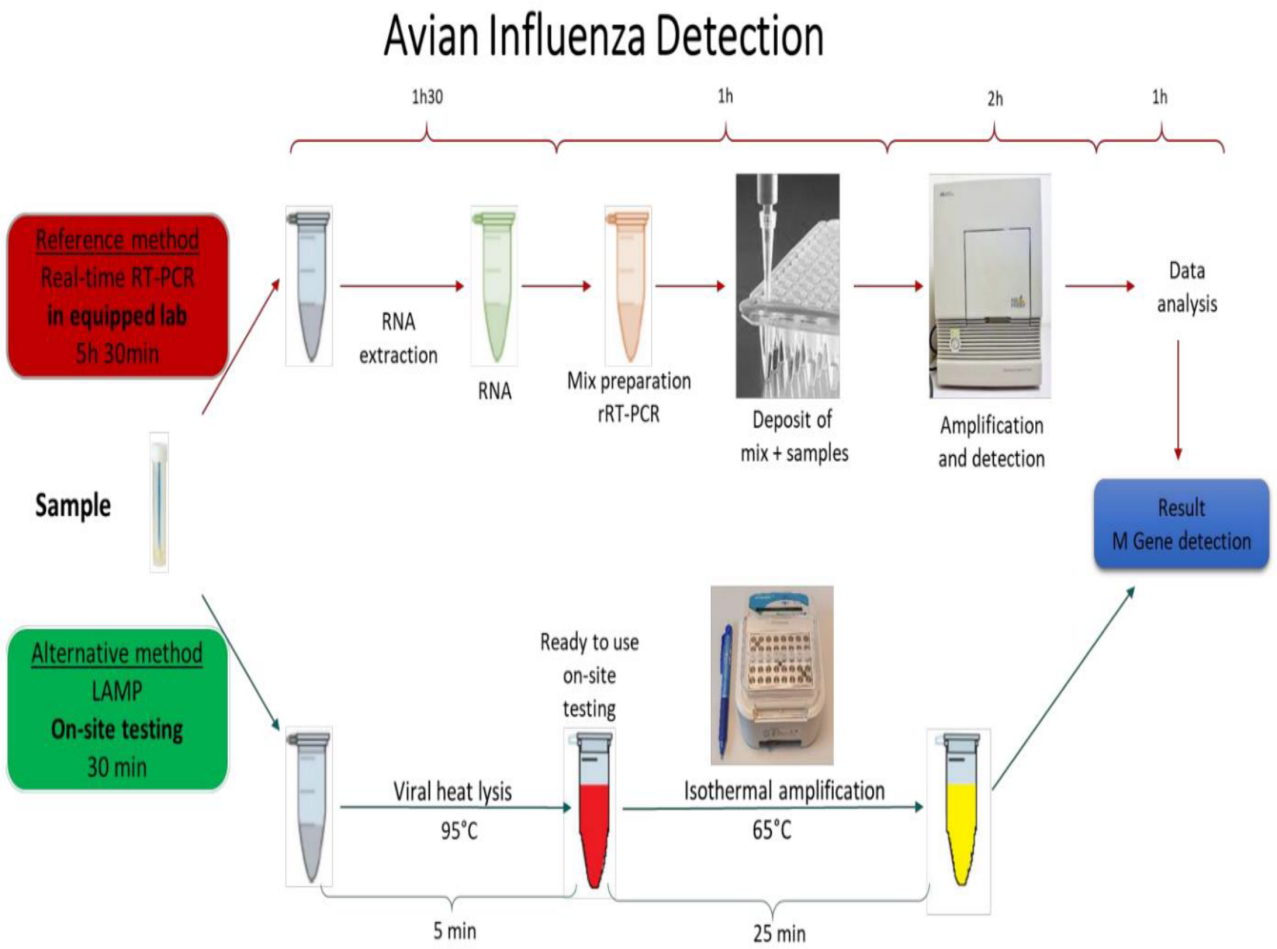
The AIV detection procedure with the reference methods (real-time RT-PCR) vs On-site testing method. The reference method is performed in equipped laboratories and takes more than 5 hours to be completed. The developed On-site testing can be done everywhere, and takes 30 minutes from sample to result.

Comparing the results of rRT-LAMP and rRT-PCR (**Figure 2**) clearly reveals that distinguishing positive and negative curves are much easier in rRT-LAMP than in rRT-PCR due to accelerated amplification in LAMP. This effect is more significant when testing samples with low target concentration that normally become positive very late in the rRT-PCR method. According to the reference protocol of RT-PCR for AIV Diagnostic Manual instructions, if the Ct value of the standard rRT-PCR is less than 30, that indicates a strong positive and high virus concentration. The samples yielding Ct values of 35-37 need to be re-tested, and any Ct value of above 36 is considered as negative if the amplification plot is linear. In rRT-PCR, the results need an extra data analysis step including a precise baseline correction to distinguish the real and false positive curves in case of samples with low target concentration. In contrast, in RT-LAMP, accelerated amplification results in a large amount of DNA amplified products. Therefore, the positive amplification curves are sharp and well distinguishable from negative results even at low concentration of the target template. This great advantage makes rRT-LAMP an interesting alternative method to the current standard rRT-PCR method. In nucleic acid amplification reactions such as PCR or LAMP, it has been shown that the specificity of the oligonucleotide primers to the target gene determines the level of efficiency of the assay (Yoda et al., 2007). Primer mismatches, although amplification can tolerate some, can severely reduce priming and the assay efficiency (Stadhouders et al., 2010). The rRT-PCR requires only a pair of primers and a probe sequence. In contrast, LAMP employs 3 to 4 primer pairs targeting 6 to 8 different regions within a small sequence (<250 bp) of the target gene. Due to genetic drift in AIV, it is a challenge to design a general pair of primers and probes to detect all AIV subtypes. The M-gene of AIV is the most conserved gene amongst AIV species and is the target gene of the rRT-PCR reference method to detect all AIV subtypes. However, our primary bioinformatics analysis revealed that there are not enough conserved regions on the AIV M gene to design an efficient LAMP primer set if the M gene sequence of AIV strains isolated from all over the world are included in the analysis. Shivakoti et al. reported challenges in identifying conserved regions on the AIV M gene for designing LAMP primer when viral sequences from Eurasian and American lineages are included in single alignment (Shivakoti et al., 2010). The challenge of designing LAMP primers against influenza virus are also reported by other researchers (Tun et al., 2014; Kim et al., 2016; Ahn et al., 2019). Therefore, we chronologically and geographically confined our input data and selected M gene sequences of AIVs isolated in the *EU* countries in the last 10 years (from 2010 upward) for designing the LAMP primer set. Several researchers have developed RT-LAMP to detect a specific subtype of AIV by targeting Hemagglutinin or Neuraminidase genes (Ge et al., 2013; Zhang et al., 2013; Bao et al., 2014; Liu et al., 2014; Luo et al., 2015; Wang et al., 2016; Hu et al., 2017; Furuyama et al., 2018). Ahn et al. designed a series of LAMP primers set for detecting various human influenza viruses in a rapid and simple colorimetric detection platform. The authors designed various LAMP primers for detecting HA gene in type A influenza and NA gene in type B influenza viruses in separate assays with sensitivity comparable to rRT-PCR. However, a multiplex test is needed in parallel experiment to detect the targeted subtypes of influenza virus in a sample (Ahn et al., 2019). On the other hand, there are few studies on the development of the LAMP method for detection of all AIV subtypes by targeting the M gene (Shivakoti et al., 2010; Yoshida et al., 2011; Tun et al., 2014; Kim et al., 2015; SHI et al., 2019). Lin et al. developed a LAMP test targeting the M gene for rapid detection of AIV in clinical specimens. The authors claimed that the designed LAMP primer was specific and was able to detect 16 AIV strains that belong to various H subtypes. However, the approach to define the conserved location on the AIV M gene for designing these LAMP primers was not clearly described. In addition, the method required RNA extract from the sample that prolonged the sample to result time (SHI et al., 2019). Tun et al. developed a one-step RT-LAMP assay for the detection of influenza A viruses by targeting the M gene with similar sensitivity compared to rRT-PCR. Similar to Lin et al. this method also required extraction of RNA from the sample. In addition, the authors suggested further studies to simplify the viral RNA extraction method for further field application and to enhance sensitivity of the assay (Tun et al., 2014). Shivakoti et al. developed a RT-LAMP assay reaching the sensitivity of 10^5^ fold dilution of the initial AIV titer, approximately 10^3^ EID_50_/reaction with a detection time of about 27-41 minutes (Shivakoti et al., 2010). The LAMP primer set, designed in this study, could successfully detect all reference AIV strains belonging to nine different AIV subtypes as well as 14 AIV strains isolated from wild birds in Denmark. The sensitivity of the optimized rRT-LAMP and on-site testing methods are compared with the standard rRT-PCR method. The sensitivity of all three methods are the same when the purified RNA samples were used as a target that was around 10^2.5^ EID_50_/mL of sample. RNA extraction and purification from samples was an essential step to obtain sensitive results in rRT-PCR assays. This additional step makes it difficult to adapt an on-site or portable device based on the PCR method because the extraction process is a troublesome procedure requiring specific equipment (Kaneko et al., 2007). In this study, a heat treatment method was employed as an alternate approach. Heat treatment - a simple method for sample preparation was reported by Nie et al. for Human *Enterovirus* detection (Nie et al., 2012). It was also employed for releasing AIV genomic RNA from viral capsid protein to be detected by LAMP (Tang et al., 2016). The RNA extraction process is costly, a multistep and time-consuming process that is difficult to adapt as a part of on-site testing assay. Alternative RNA isolation processes such as magnetic beads or silica gel are also not ideal and easily implementable for on-site testing. In contrast to the conventional RNA extraction process, the heat treatment approach is rapid, simple, and single step. It is easy to perform and to adapt for development of a simplified protocol for on-site testing. As shown in **Scheme 1**, employing a heat treatment process will shorten the sample to result time from more than five hours (for rRT-PCR) to 30 min (for rRT-LAMP) and simplifies the whole process making it suitable for on-site testing.

As frequently reported, LAMP is more resistant to inhibitors than PCR, especially in the complex background matrix such as clinical samples (Kaneko et al., 2007; Tani et al., 2007; Nie et al., 2012; Nyan et al., 2014; Damhorst et al., 2015; Lee et al., 2016a; Lee et al., 2016b). This feature of LAMP simplifies the sample preparing steps for pathogen detection in real samples. To evaluate the feasibility of detecting AIV in a real matrix sample with a simplified sample preparation step, matrices such as oral and cloacal swabs from live birds as well as other organ samples were collected and tested. Oral and cloacal swab sampling from live birds is the proper method of sampling for AIV detection in a live bird. However, highly pathogenic avian influenza viruses replicate in multiple tissues and lead to high mortality in chickens (Yoshida et al., 2011). Therefore, detection of AIV in the birds’ organ samples is of greater interest in special cases such as in wild dead birds or in monitoring poultry carcass in slaughterhouses. In the presence of high target concentration, the effect of the background matrix on the real-time RT-LAMP amplification curve was insignificant (**Figure 4A**). Although the effect of the matrix is visible when the concentration of the target is low (**Figure 4B**), this did not have any adverse effect on the result as well as on the sensitivity of the assay. Therefore, even a complex background matrix such as chicken organ samples can be directly added to the rRT-LAMP test after heating treatment. This has a great advantage on the development of bioanalytical assays because it omits the cumbersome RNA purification step and makes the total process faster, low cost, and compatible for on-site testing. The on-site test developed in this study was an end-point detection test therefore no effect of the background matrix was observed in the samples with both high and low target concentration. However, the on-site test approach was not compatible with two organ samples including pancreas and lung, as there was a change in the color of the reaction mixture upon addition of the sample (**Figure 4C**). In the On-site test, the principle of visual detection is based on the change in the pH during LAMP reaction. Hydrogen ion is a byproduct of DNA amplification that reduces the pH of the weakly buffered on-site LAMP reaction mixture. The On-site test LAMP mixture contains a pH indicator, phenol red, that shifts its original color from pink to yellow when the pH drops from >8.2 to <6.8. Independent of the presence of an RNA template, some clinical samples may lead to acidification of the weakly buffered reaction mix (Dao Thi et al., 2020). With he exception of these two organ samples, the on-site test successfully detected AIV in all the matrix samples with the sensitivity as low as 10^0.8^ EID_50_ per reaction within 30 including sample preparation.

## Conclusion

In conclusion, in this study we described a newly developed rRT-LAMP with a unique designed primer set based on genome sequences of recently isolated AIV. The method is sensitive, specific to detect all AIV. The method is very rapid, cost effective and poses potential for on-site testing for rapid screening of AIV in poultry production, in wild birds as well as in diagnosis.

## Data Availability Statement

The original contributions presented in the study are included in the article/supplementary materials. Further inquiries can be directed to the corresponding author.

## Author Contributions

MG performed bioinformatics analysis and designed the primers. BG and MF prepared AIV inactivated strains. MG and TQ performed the experiments. MG, AW, DB, and AV analyzed data and interpreted the results. MG and DB wrote the first draft manuscript. All authors read and approved the final version of the manuscript.

## Funding

This research was funded by the European Union’s Horizon 2020 research and innovation program *via* the VIVALDI project, grant agreement No: 773422

## Conflict of Interest

The authors declare that the research was conducted in the absence of any commercial or financial relationships that could be construed as a potential conflict of interest.
